# Reproducibility and Validity of a Self-administered Food Frequency Questionnaire Used in the JACC Study

**DOI:** 10.2188/jea.15.S9

**Published:** 2005-05-16

**Authors:** Chigusa Date, Mitsuru Fukui, Akio Yamamoto, Kenji Wakai, Azusa Ozeki, Yutaka Motohashi, Chieko Adachi, Naoyuki Okamoto, Michiko Kurosawa, Yuko Tokudome, Yoko Kurisu, Yoshiyuki Watanabe, Kotaro Ozasa, Shuichi Nakagawa, Noritaka Tokui, Takesumi Yoshimura, Akiko Tamakoshi

**Affiliations:** 1 Department of Nutrition and Food Sciences, Faculty of Human Environmental Sciences, Mukogawa Women’s University.; 2 Laboratory of Statistics, Osaka City University Medical School.; 3 Infectious Disease Research Division, Hyogo Prefectural Institute of Public Health and Environmental Sciences.; 4 Aichi Cancer Center Research Institute.; 5 Yakumo-cho town office.; 6 Akita University School of Medicine.; 7 Showa University School of Medicine.; 8 Kanagawa Cancer Center.; 9 Juntendo University School of Medicine.; 10 Nagoya Bunri University.; 11 Gifu University Graduate School of Medicine.; 12 Kyoto Prefectural University of Medicine Graduate School of Medical Science.; 13 Nakagawa Clinic (Nephrology and Urology).; 14 Institute of Industrial Ecological Sciences, University of Occupational and Environmental Health.; 15 Fukuoka Institute of Health and Environmental Sciences.; 16 Nagoya University Graduate School of Medicine.

**Keywords:** food frequency questionnaire, weighed dietary record, validity, reproducibility

## Abstract

BACKGROUND: A self-administered questionnaire on dietary habits used in the JACC Study contained a 40-item food frequency questionnaire (FFQ). Although more than 110 thousand subjects enrolled in JACC Study and responded to the FFQ, no validation study has been conducted to date.

METHODS: Eighty-five volunteers among the cohort members completed 2 FFQs (FFQs 1&2) and 12-day weighed dietary records (WDR). The interval between the two FFQs was one year. During the one year, the subjects carried out a 3-consecutive-day WDR in each season. We tested the reproducibility by using two FFQs. Also, we tested the validity of the FFQ by using the 12-day WDR as a gold standard.

RESULTS: The intake frequencies of the 2 FFQs often agreed, showing the Spearman correlation coefficients ranging from 0.42 (edible wild plants) to 0.86 (coffee). The Spearman correlation coefficients of the energy and nutrient intakes from FFQ2, and that of the 12-day WDR were 0.20(energy) to 0.46 (animal protein, potassium). After adjusting the energy intake, the correlation coefficients showed 0.21(fish fat) to 0.51(animal fat). When classifying the FFQ2 and WDR by quartiles and examining the degree of agreement between the two methods, we obtained its median 30%.

CONCLUSIONS: The FFQ is suitable to deal with a large group of subjects. However, since the energy and the amount of nutrient intake from this FFQ can not show the overall dietary intake situation, the subjects’ dietary intake should be assessed by categories.

“The Japan Collaborative Cohort Study (JACC Study) for Evaluation of Cancer Risk Sponsored by the Ministry of Education, Science, Sports and Culture of Japan (Monbusho)” is a large-scale population-based cohort study which launched in 45 municipalities all over Japan.^1^

Epidemiologic data were collected at baseline (1988-1990) through a self-administered questionnaire with the written informed consent of the respondents, including such demographic information as sex, date of birth, marital status, number of children and medical history. Questions on their dietary habits were also included, such as the bowls of steamed rice they ate daily; the intake frequency of miso (traditional Japanese soybean paste) soup; the amount of its daily consumption in bowls at baseline. There were also questions about the intake frequency concerning 33 items with the use of 5 response categories, and those concerning alcoholic beverages and soft drinks.

In many large-scale population based on nutritional epidemiologic studies for analyzing the relation of nutrient intakes and health outcomes, a self-administered semi-quantitative food frequency questionnaire (SQFFQ) was used.^2^ SQFFQ is composed of a food list and a frequency response section in which subjects are asked to report how often they consumed foods daily. This is the method by which the daily intake of nutrients was calculated by multiplying the intake frequency by the nutrients contained in portion sizes. However, in Japan little attention has been paid so far to the validity of the method of computing the amount of nutrient intake this way^3^. Moreover, although the intake frequency of specific food items or food groups is used to clarify the relation between the frequency and the incidence of diseases, a validation study has not been conducted, based on the actual intake frequency counted from the multiple-day dietary records.

We developed the method of evaluating the frequency and amount of consumed steamed rice, miso soup and beverages, and 33 food items. We tested the reproducibility and validity of frequency response and the method of estimating the amount of nutrients.

## METHODS

### Subjects

From among 24 groups who participated in the JACC Study, we obtained 5 to 10 volunteers from the 15 groups who could conduct the weighed dietary record method, and regarded them as subjects. They came from all over Japan: from prefectures in the north to those in the south such as Hokkaido, Akita, Tokyo, Kanagawa, Yamanashi, Nagano, Aichi, Gifu, Kyoto, Hyogo, and Fukuoka. Except for Kyoto (3 groups) and Aichi and Gifu (2 groups each), there was one group from such prefectures. The participants numbered 89 when the research started. Here are the specific numbers of the subjects and their prefectures: 5, Yakumo Town, Hokkaido; 5, Omori Town, Akita; 5, Samukawa Town, Kanagawa; 10, Higashi-Yamanashi County, Yamanashi; 6, Shirakawa Town, Gifu; 5, Gifu City, Gifu;4, Wachi Town, Kyoto; 7, Wazuka Town, Kyoto; 5, Keihoku Town, Kyoto; 10, Ichinomiya Town, Hyogo; 5, Saikawa Town, Fukuoka. In addition, as occupational groups, 11 from preventive medicine associations in Tokyo; 5 personnel from the Nagoya City government and 5 dietitians from Nagoya City also participated, totaling of 89 subjects.

### Research Design

**Figure 1.**
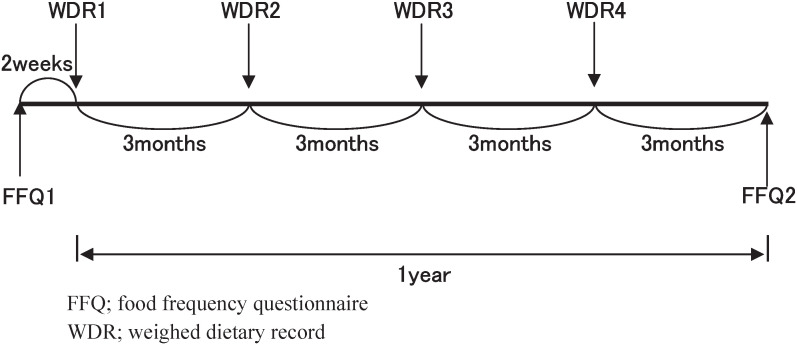
Research design of JACC Study food frequency questionnaire validation study.

The research design is outlined in Figure 1. As a method of showing the usual dietary intake level, we adopted the weighed dietary record method (WDR) by which the subjects recorded the dietary intake for 3 consecutive days (week days) every 3 months: spring, summer, autumn and winter. In other words, the subjects did the WDR, totally 12 days a year. Owing to the various stages of preparation for the research, the first group started in the summer of 1997, and the last one in the summer of 1998. As a result, the WDR was finished in all the areas by the spring of 1999. The first self-administered food frequency questionnaire (FFQ1) was carried out 2 weeks before the first WDR, and the second FFQ (FFQ2) took place 3 months after the fourth WDR. The interval between FFQs 1 &2 was one year.

Because this is a multi-center research study, the standardization of the method was necessary. Therefore, those in charge of the dietary survey in every group met together before the research started to receive detailed instructions about the data coding system and the research procedures. Because both food and cooking names were given in the food item section in the questionnaire, we decided to code both of them in order to compute nutrient contents, recipe by recipe.

The subjects were provided with a digital cooking scale (TANI-TA No. 1141, Tokyo). After the research, the cooking scales were given to them as a token of our appreciation of their cooperation.

### Data Collection

When the WDR was finished for each season, the person in charge of the dietary survey collected the recording sheets from WDR. Each group’s dietitian codified food items and food weights. Those data were mailed out to the dietary survey center in Osaka, where the dietitian in the dietary survey center checked the contents of the input data. If she found something ambiguous or unprocessed, she contacted the person in charge for clarification, thus completing the processing of the appropriate data. The self-administered FFQs 1 & 2 were sent to Nagoya, the center of the JACC Study. After they were processed at Nagoya University, the FFQ data were mailed to the dietary survey center.

### Computation of Nutrient Intakes from WDR

Based on the Standard Tables of Food Composition in Japan fourth revised edition,^4^ and the fatty acid composition table of Japanese food,^5^ we calculated the amounts of energy, protein (animal and vegetable), fat (animal, vegetable and fish), iron, calcium, potassium, salt, vitamin A, vitamin B_1_, vitamin B_2_, vitamin C, total fatty acid, saturated fatty acid (SFA), monounsaturated fatty acid (MUFA), polyunsaturated fatty acid (PUFA), and cholesterol. However, since the table has no entries for individual fatty acid components, we obtained a fatty acid composition database from Nagoya City University (Professor S. Tokudome)^6^ and from the National Cancer Center Research Institute East (Dr. S. Sasaki).^7^

### Computation of Nutrient Intakes from FFQ

The content of FFQs used in the research are as follows: the number of bowls of steamed rice consumed daily at baseline; the intake frequency of miso soup; and the number of bowls a day at baseline; questions about the intake frequency of 33 kinds of food items, with 5 multiple choice responses: almost never; 1-2 times a month; 1-2 times a week; 3-4 times a week; almost everyday. There were other questions about the drinking habits of alcoholic beverages and soft drinks: coffee, black tea, Japanese green tea and Chinese tea. The questionnaires used in this study are shown in Appendix1 and Appendix2.

Each frequency weight was set at 0, 0.05 (1.5/30), 0.214 (1.5/7), and 0.5 (3.5/7), with 1.0 signifying the response ‘almost everyday’. As for the frequency of miso soup, we set the four frequency weights, (i.e., every day; every other day; several times a month; seldom) as 1.0, 0.5, 0.1, and 0, respectively.

With respect to the intake amount of consumed steamed rice, replies were made in terms of the number of medium-sized bowls consumed. Hence, one such bowl was estimated to contain 140 g. The amount was thus obtained by multiplying 140 g by the number of bowls. The nutrient value of steamed rice is based on the Standard Tables of Food Composition in Japan, 4th revised edition.^4^

As for other foods, there were no questions about portion sizes in the questionnaires. However, we estimated them from the WDR results. That is, we computed the daily amount of food eaten by each person, based on the data from WDR, and determined the median as portion sizes (Table 1). As for food items without any specification, we decided on their nutrient composition as follows: there was no mention of the kinds of beef (e.g., sirloin, fillet) in the questionnaire, although various parts of beef were referred to in WDR. We selected them and computed those weighted mean ingredient values, based on the Standard Tables of Food Composition in Japan, 4th revised edition.^4^ The nutrient composition of foods other than beef was calculated in the same way.

**Table 1.** Portion sizes for 33 food items.

Foods /Beverages	portion size(g)
Beef	36
Pork (excluding ham or sausage)	41
Ham or sausage	22
Chicken	43
Liver	53
Eggs	37
Milk	146
Yogurt	98
Cheese	17
Butter	5
Margarine	6
Deep-fried foods or tempura	113
Fried vegetables	112
Fresh fish	63
Kamaboko (fish paste)	20
Dried fish or salted fish	29
Spinach or garland chrysanthemum	42
Carrot or pumpkin	23
Tomatoes	63
Cabbage or head lettuce	40
Chinese cabbage	66
Edible wild plants	36
Fungi (enokidake, shiitake mushroom)	17
Potatoes	52
Algae (seaweeds)	7
Pickles	26
Preserved foods using soy sause	10
Boiled beans	40
Tofu (soybean curd)	60
Citrus fruits	68
Fresh fruits juice (in summer)	160
Fruits (excluding citrus variety)	93
Sweets	38

As for butter and margarine, we calculated the median portion size by the same method as mentioned above. In Japan, few people use butter or margarine for cooking. Instead, they spread it on toast. We calculated the median portion size of bread, which turned out be 60 g. We added its nutrient value to the nutritive composition of butter and margarine.

When any particular item was used as a recipe in the questionnaire (such as deep fried foods or tempura, fried vegetables, pickles, preserved foods using soy sauce, boiled beans, etc.), we computed the median daily amount of cooking based on WDR by selecting the code in the names. The nutrient composition value was computed, based on the weighted mean value of all foods used for such cooking.

We calculated the intake amount of coffee, black tea, Japanese green tea and Chinese tea by multiplying 100 g per cup by the number of cups. For their nutrient values, we used the coffee infusion, black tea infusion, coarse-grade Japanese tea infusion and oolong tea infusion as listed in the Standard Tables of Food Composition in Japan, 4th revised edition.^4^ In the questions regarding coffee and black tea, if sugar and milk were taken for drinking either beverage, we added 4 g for the sugar, 50 g for the milk and 2.5 g for substitute cream, each of which is the median amount used per cup in WDR. Then we calculated the sum of the products of the frequency weight and the nutrient content of the portion size for each food as the daily nutrient intake.

### Data Analysis

We tested the reproducibility of intake frequency from FFQs 1 & 2 and the amounts of the various nutrients calculated from FFQs 1 & 2 by the Spearman rank correlation coefficient between the two FFQs, which were carried out twice at the interval of one year.

In order to test the validity of the intake frequency of FFQ2, we defined the gold standard of intake frequency based on WDR as follows. First, corresponding to the frequency category of FFQ (almost never; 1-2 times a month; 1-2 times a week; 3-4 times a week; almost every day), we classified the frequency of 12-days WDR into five categories: never; 1 to 2 days; 3-5 days; 6-11 days; 12 days. Next, we counted the number of days during which the subjects ate each of the 33 food items, steamed rice, miso soup and beverages during the 12-day WDR and thereby determined the corresponding frequency categories. Then, we computed the Spearman rank correlation coefficients of 5 category classifications of the WDR and the FFQ2.

In order to examine the validity of the amount of nutrient intake computed from FFQ2 as mentioned above, we computed the Spearman correlation coefficients (both crude correlation coefficients, and energy adjusted correlation coefficients^9^) between the WDR and the FFQ2.

We used the software package (SPSS^®^ 11.5J for Windows, SPSS Inc.) for computation.

## RESULTS

### Analysis Subjects

Though unfortunately nine participants dropped out of the research, the remaining 85 persons completed the two FFQs (FFQ1 & FFQ2) and the 12-day WDR, and they were regarded as analysis subjects. Detailed information on them is shown in Table 2-1,2-2.

**Table 2-1.** The number of analysis subjects by group and sex.

Group	Prefecture	Female	Male
Yakumo Town	Hokkaido	4	1
Omori Town	Akita	5	0
Samukawa Town	Kanagawa	5	0
Higashi-Yamanashi County	Yamanashi	10	0
Shirakawa Town	Gifu	5	0
Gifu City	Gifu	4	0
Wachi Town	Kyoto	5	0
Wazuka Town	Kyoto	7	0
Keihoku Town	Kyoto	5	0
Ichinomiya Town	Hyogo	10	0
Saikawa Town	Fukuoka	5	0
Tokyo preventive medicine association	Tokyo	3	6
Personnel from Nagoya City government	Aichi	4	1
Dietitians from Nagoya City	Aichi	5	0

Total		77	8

**Table 2-2.** Age distribution of analysis subjects.

Age(year)	Number	%
20-29	1	1.2
30-39	8	9.4
40-47	25	29.4
50-59	31	36.5
60-69	19	22.4
70-79	1	1.2

Total	85	100

### Intake Frequency Assessment

In Table 3, the Spearman rank correlation coefficients between FFQ1 & FFQ2 are shown, in the order of height. Coffee had the highest correlation coefficient (0.86), whereas edible wild plants the lowest (0.42). The correlation coefficients for all the foods were found to be statistically significant, and the mean and the median of 39 food items were 0.60.

**Table 3.** Spearman rank correlation coefficients between two frequencies (five categories) assessed with food frequency questionnaire administered twice apart one year. (n=88)

Foods/Beverages	Spearman correlation coefficient*
Coffee	0.86
Black tea	0.74
Miso soup	0.72
Liver	0.71
Milk	0.69
Pickles	0.68
Ham or sausage	0.67
Eggs	0.66
Cabbage or head lettuce	0.66
Algae (seaweeds)	0.66
Tofu (soybean curd)	0.64
Margarine	0.63
Spinach or garland chrysanthemum	0.63
Chinese cabbage	0.63
Sweets	0.62
Japanese green tea	0.62
Steamed rice	0.62
Potatoes	0.61
Chinese tea	0.61
Fungi (enokidake, shiitake mushroom)	0.60
Beef	0.59
Boiled beans	0.59
Fruits (excluding citrus variety)	0.58
Cheese	0.57
Kamaboko (fish paste)	0.57
Citrus fruits	0.57
Preserved foods using soy sause	0.56
Butter	0.55
Fresh fruits juice(in summer)	0.55
Yogurt	0.54
Fresh fish	0.54
Chicken	0.53
Tomatoes	0.53
Deep-fried foods or tempura	0.52
Fried vegetables	0.46
Dried fish or salted fish	0.46
Pork (excluding ham or sausage)	0.44
Carrot or pumpkin	0.43
Edible wild plants	0.42

Mean	0.60
Median	0.60

* : If r=0.34 then p=0.001 (n=85).

In Table 4, the Spearman rank correlation coefficients between FFQ2 and 12-day WDR are shown, in the order of height. Coffee was found to have the highest (0.81) correlation coefficient. while edible wild plants the lowest (0.07). This order was the same as that of reproducibility. The mean was 0.39 and the median was 0.42. The foods whose correlation coefficients were not significant were deep-fried foods or tempura, preserved foods using soy sauce, kamaboko (fish paste), fried vegetables, and edible wild plants.

**Table 4.** Spearman rank correlation coefficients between two frequencies (five categories) assessed with 2nd food frequency questionnaire and weighed dietary record for 12 days. (n=85)

Foods/Beverages	Spearman correlation coefficient*
Coffee	0.81
Miso soup	0.69
Milk	0.65
Ham or sausage	0.63
Steamed rice	0.63
Margarine	0.62
Black tea	0.60
Yogurt	0.58
Fungi (enokidake, shiitake mushroom)	0.54
Butter	0.51
Tofu (soybean curd)	0.50
Beef	0.49
Algae (seaweeds)	0.46
Fresh fish	0.45
Tomatoes	0.45
Pickles	0.45
Chicken	0.44
Cheese	0.44
Chinese tea	0.43
Spinach or garland chrysanthemum	0.42
Eggs	0.40
Fruits (excluding citrus variety)	0.39
Pork (excluding ham or sausage)	0.37
Sweets	0.36
Cabbage or head lettuce	0.33
Carrot or pumpkin	0.32
Boiled beans	0.30
Citrus fruits	0.26
Fresh fruits juice(in summer)	0.24
Potatoes	0.23
Japanese green tea	0.21
Liver	0.20
Chinese cabbage	0.18
Deep-fried foods or tempura	0.17
Dried fish or salted fish	0.17
Preserved foods using soy sause	0.16
Kamaboko (fish paste)	0.12
Fried vegetables	0.11
Edible wild plants	0.07

Mean	0.39
Median	0.42

* : If r=0.18, then p=0.05; r=0.25, then p=0.01; and r=0.34, then p=0.001 (n=85).

**Figure 2.**
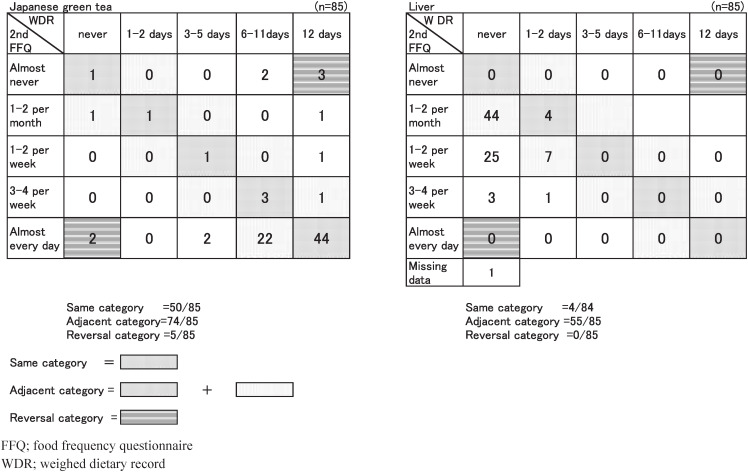
Contingency tables for joint classification intake frequency assessed by 2nd food frequency questionnaire and weighed dietary record for 12 days.

Figure 2 shows the relation between the frequency of Japanese green tea consumption and liver reported in the FFQ2, and their frequency evaluated in the 12-day WDR. We show the results by dividing them into three frames: the one where the frequency categories of the two methods corresponded, the one where the frequency categories are adjacent to each other, and the one where the frequency categories were in reversely results.

Table 5 shows the percentages classified into the same category according to the two methods shown in Figure 2, the percentage classified into the same category and the contiguity category, and the percentage classified into the reversely categories, in the descending order within the same category. The mean percentage of those classified into the same category was 36%. Japanese green tea (59%) was the highest, whereas liver (any kinds) (5%) was the lowest. The classifications into reversely categories were found to exist in Japanese green tea (6%), Chinese tea (2%), milk, cheese, tofu, sweets, Chinese cabbage, and boiled beans (1%).

**Table 5.** Comparison of 2nd food frequency questionnaire with weighed dietary record for 12 days for frequency on joint classification by five categories. (%)

Foods /Beverages	Same category	Adjacent category	Reversal category
Japanese green tea	59	87	6
Chinese tea	57	84	2
Milk	48	87	1
Black tea	48	84	0
Chicken	47	95	0
Beef	45	96	0
Pork (excluding ham or sausage)	44	98	0
Butter	43	85	0
Margarine	43	81	0
Carrot or pumpkin	42	92	0
Algae (seaweeds)	40	94	0
Coffee	40	80	0
Fried vegetables	40	76	0
Fresh fish	39	93	0
Cheese	38	86	1
Eggs	37	95	0
Cabbage or head lettuce	37	90	0
Ham or sausage	37	88	0
Tofu (soybean curd)	36	90	1
Fungi (enokidake, shiitake mushroom)	35	93	0
Kamaboko (fish paste)	35	83	0
Pickles	34	82	0
Deep-fried foods or tempura	34	81	0
Preserved foods using soy sause	33	73	0
Fruits (excluding citrus variety)	32	90	0
Sweets	30	80	1
Dried fish or salted fish	30	79	0
Citrus fruits	30	76	0
Fresh fruits juice (in summer)	29	60	0
Spinach or garland chrysanthemum	27	86	0
Tomatoes	27	82	0
Chinese cabbage	27	72	1
Potatoes	26	88	0
Yogurt	24	70	0
Boiled beans	24	66	1
Edible wild plants	17	58	0
Liver	5	65	0

Mean	36	83	
Median	36	84	

### Nutrient Intake Assessment

Table 6 shows mean total energy and 11 nutrients intakes assessed with WDR and FFQ. Also, Table 7 shows the amount of fatty acid intake assessed with WDR and FFQ. The intake amount of energy and nutrients assessed with FFQ was lower than with WDR. The percentage difference{ (FFQ − WDR) / WDR } changed between −64% of salt and −15% of vitamin A. The mean percentage difference was −37% and, the median was −36% among nutrients.

**Table 6.** Energy and nutrient intakes assessed with weighed dietary record (WDR) for 12-days and food frequency questionnaire (FFQ).

(n=85) Nutrient		unit	Weighed dietary record (WDR) for 12-days					Food frequency questionnaire (FFQ)					% difference^†^
	
			Mean	±	SD	*Median*	(25%, 75%)*	Mean	±	SD	*Median*	(25%, 75%)*	
Energy		kcal/day	1792	±	285	*1722*	*(1593, 1996)*	1193	±	234	*1201*	*(1038, 1366)*	-33

Total protein	Crude	g/day	75.3	±	13.0	*76.0*	*(67.1, 82.4)*	48.1	±	13.0	*46.7*	*(38.0, 56.2)*	-36
Energy adjusted			75.3	±	7.4	*75.1*	*(70.1, 79.3)*	48.1	±	6.6	*48.5*	*(44.7, 51.6)*	-36

Animal protein	Crude	g/day	40.9	±	10.4	*41.3*	*(34.1, 46.7)*	25.7	±	9.6	*25.6*	*(17.6, 32.3)*	-37
Energy adjusted			40.9	±	7.7	*40.7*	*(36.4, 46.2)*	25.7	±	6.7	*26.4*	*(22.6, 29.2)*	-37

Total fat	Crude	g/day	53.2	±	12.0	*52.8*	*(45.9, 60.7)*	29.6	±	9.0	*28.6*	*(23.5, 34.8)*	-44
Energy adjusted			53.2	±	6.8	*53.6*	*(49.0, 57.4)*	29.6	±	5.2	*29.5*	*(26.6, 32.9)*	-44

Animal fat	Crude	g/day	20.8	±	7.2	*19.9*	*(15.4, 25.4)*	14.5	±	5.5	*14.8*	*(10.4, 18.0)*	-30
Energy adjusted			20.8	±	5.6	*20.3*	*(16.6, 25.1)*	14.5	±	3.9	*14.9*	*(11.6, 16.9)*	-30

Vegetable fat	Crude	g/day	26.3	±	6.6	*25.7*	*(22.4, 29.8)*	11.7	±	3.5	*11.5*	*(9.3, 14.0)*	-56
Energy adjusted			26.4	±	5.4	*26.9*	*(23.5, 29.9)*	11.7	±	2.4	*11.9*	*(10.7, 13.0)*	-56

Fish fat	Crude	g/day	6.0	±	3.1	*5.9*	*(3.7,7.6)*	3.4	±	3.0	*3.0*	*(1.8, 4.8)*	-43
Energy adjusted			6.0	±	2.8	*6.3*	*(4.3, 8.2)*	3.4	±	1.8	*3.6*	*(2.3, 4.89)*	-43

Iron	Crude	mg/day	11.3	±	2.2	*11.2*	*(9.5, 12.8)*	7.0	±	2.1	*6.4*	*(5.4, 8.4)*	-38
Energy adjusted			11.3	±	1.9	*11.2*	*(9.8, 12.7)*	7.0	±	1.4	*6.9*	*(6.1, 7.9)*	-38

Calcium	Crude	mg/day	663	±	191	*632*	*(542, 745)*	496	±	151	*485*	*(391, 602)*	-25
Energy adjusted			663	±	171	*642*	*(560, 749)*	496	±	100	*512*	*(433, 551)*	-25

Potassium	Crude	mg/day	3000	±	675	*2983*	*(2433, 3343)*	1939	±	552	*1948*	*(1529, 2248)*	-35
Energy adjusted			3000	±	569	*2881*	*(2632, 3378)*	1939	±	371	*1996*	*(1719, 2156)*	-35

Salt	Crude	g/day	10.9	±	2.5	*10.7*	*(9.2, 12.0)*	4.0	±	1.7	*3.7*	*(2.9, 4.6)*	-64
Energy adjusted			10.9	±	2.0	*10.6*	*(9.4, 11.7)*	4.0	±	1.2	*3.9*	*(3.4, 4.6)*	-64

Vitamin A	Crude	IU/day	2909	±	1339	*2583*	*(2040, 3649)*	2477	±	1400	*2335*	*(1476, 2939)*	-15
Energy adjusted			2909	±	1222	*2616*	*(2154, 3433)*	2477	±	1271	*2342*	*(1583, 3107)*	-15

Vitamin B_1_	Crude	mg/day	1.08	±	0.26	*1.07*	*(0.88, 1.21)*	0.71	±	0.20	*0.69*	*(0.54, 0.85)*	-35
Energy adjusted			1.08	±	0.20	*1.06*	*(0.95, 1.15)*	0.71	±	0.12	*0.71*	*(0.64, 0.76)*	-35

Vitamin B_2_	Crude	mg/day	1.53	±	0.34	*1.54*	*(1.30, 1.76)*	1.05	±	0.31	*1.06*	*(0.82, 1.28)*	-31
Energy adjusted			1.53	±	0.24	*1.54*	*(1.37, 1.69)*	1.05	±	0.21	*1.08*	*(0.96, 1.17)*	-31

Vitamin C	Crude	mg/day	138	±	43	*136*	*(108, 165)*	96	±	38	*93*	*(68, 121)*	-30
Energy adjusted			138	±	41	*132*	*(106, 158)*	96	±	31	*95*	*(76, 118)*	-30

Cholesterol	Crude	mg/day	378	±	95	*374*	*(323, 443)*	228	±	87	*236*	*(153, 297)*	-40
Energy adjusted			378	±	80	*381*	*(329, 418)*	228	±	65	*227*	*(181, 276)*	-40

* : 25 and 75 percentiles.

† : (FFQ mean-WDR mean)/WDR mean (%)

**Table 7.** Fatty acid intake assessed with weighed dietary record (WDR) for 12-days and food frequency questionnaire (FFQ).

(n=85) Fatty acid		unit	Weighed dietary record (WDR) for 12-days					Food frequency questionnaire (FFQ)					% difference^†^
	
			Mean	±	SD	*Median*	(25%, 75%)*	Mean	±	SD	*Median*	(25%, 75%)*	
TFA^‡^	Crude	mg/day	53.2	±	12.0	*52.6*	*(45.7,61.0)*	29.6	±	9.0	*28.6*	*(23.4,34.9)*	-44
Energy adjusted			53.2	±	6.8	*52.9*	*(48.8,57.5)*	29.6	±	5.2	*29.8*	*(26.3,32.8)*	-44

SFA^§^	Crude	mg/day	14.7	±	4.0	*14.4*	*(11.8,17.2)*	9.5	±	3.2	*9.3*	*(7.1,11.5)*	-35
Energy adjusted			14.7	±	2.6	*15.0*	*(12.7,16.6)*	9.5	±	2.0	*9.5*	*(8.3,10.9)*	-35

Myristic (14:0)	Crude	mg/day	17.6	±	4.4	*17.0*	*(14.2,20.7)*	8.9	±	2.9	*8.7*	*(6.7,10.6)*	-49
Energy adjusted			17.6	±	2.7	*17.9*	*(15.7,19.4)*	8.9	±	1.8	*9.1*	*(7.5,9.9)*	-49

Palmitic (16:0)	Crude	mg/day	13.2	±	2.9	*12.8*	*(11.5,14.5)*	6.6	±	2.0	*6.2*	*(5.4,7.5)*	-50
Energy adjusted			13.2	±	2.0	*13.1*	*(12.2,14.1)*	6.6	±	1.2	*6.7*	*(5.9,7.5)*	-50

Stearic (18:0)	Crude	mg/day	1.2	±	0.5	*1.2*	*(0.8,1.5)*	0.9	±	0.4	*0.9*	*(0.7,1.1)*	-26
Energy adjusted			1.2	±	0.4	*1.2*	*(1.0,1.4)*	0.9	±	0.3	*0.9*	*(0.7,1.1)*	-26

MUFA^||^	Crude	mg/day	8.6	±	2.1	*8.3*	*(7.0,9.8)*	5.4	±	1.6	*5.3*	*(4.1,6.4)*	-37
Energy adjusted			8.6	±	1.3	*8.7*	*(7.4,9.6)*	5.4	±	1.0	*5.3*	*(4.8,6.1)*	-37

Palmitoleic (16:1)	Crude	mg/day	1.0	±	0.3	*0.9*	*(0.8,1.1)*	0.6	±	0.2	*0.6*	*(0.4,0.7)*	-41
Energy adjusted			1.0	±	0.2	*1.0*	*(0.8,1.1)*	0.6	±	0.1	*0.6*	*(0.5,0.7)*	-41

Oleic (18:1)	Crude	mg/day	3.3	±	0.9	*3.3*	*(2.7,3.8)*	2.0	±	0.7	*2.0*	*(1.5,2.4)*	-41
Energy adjusted			3.3	±	0.6	*3.4*	*(2.9,3.7)*	2.0	±	0.4	*1.9*	*(1.7,2.3)*	-41

PUFA^¶^	Crude	mg/day	15.4	±	3.9	*14.9*	*(12.4,17.9)*	7.8	±	2.5	*7.6*	*(6.0,9.2)*	-49
Energy adjusted			15.4	±	2.5	*15.6*	*(13.6,17.0)*	7.8	±	1.5	*7.8*	*(6.7,8.9)*	-49

n-3 PUFA	Crude	mg/day	10.0	±	2.2	*9.7*	*(8.6,10.9)*	5.0	±	1.5	*4.9*	*(4.1,5.8)*	-49
Energy adjusted			10.0	±	1.7	*9.9*	*(9.1,10.9)*	5.0	±	0.9	*5.1*	*(4.5,5.7)*	-49

Linolenic (18:3n-3)	Crude	mg/day	1.7	±	0.5	*1.6*	*(1.4,1.9)*	0.7	±	0.3	*0.7*	*(0.6,0.8)*	-56
Energy adjusted			1.7	±	0.4	*1.7*	*(1.5,1.9)*	0.7	±	0.2	*0.8*	*(0.6,0.9)*	-56

EPA** (20:5n-3)	Crude	mg/day	0.03	±	0.01	*0.02*	*(0.02,0.03)*	0.02	±	0.01	*0.02*	*(0.01,0.02)*	-34
Energy adjusted			0.03	±	0.01	*0.02*	*(0.02,0.03)*	0.02	±	0.00	*0.02*	*(0.01,0.02)*	-34

DPA^† †^ (22:5n-3)	Crude	mg/day	0.2	±	0.04	*0.2*	*(0.1,0.2)*	0.1	±	0.04	*0.1*	*(0.1,0.1)*	-40
Energy adjusted			0.2	±	0.03	*0.2*	*(0.1,0.2)*	0.1	±	0.03	*0.1*	*(0.1,0.1)*	-40

DHA^‡ ‡^ (22:6n-3)	Crude	mg/day	0.4	±	0.2	*0.4*	*(0.2,0.5)*	0.2	±	0.1	*0.2*	*(0.1,0.3)*	-45
Energy adjusted			0.4	±	0.2	*0.4*	*(0.3,0.5)*	0.2	±	0.1	*0.2*	*(0.1,0.3)*	-45

n-6 PUFA	Crude	mg/day	0.1	±	0.05	*0.1*	*(0.1,0.1)*	0.1	±	0.03	*0.1*	*(0.03,0.1)*	-43
Energy adjusted			0.1	±	0.04	*0.1*	*(0.1,0.1)*	0.1	±	0.03	*0.1*	*(0.04,0.1)*	-43

Linoleic (18:2n-6)	Crude	mg/day	0.7	±	0.3	*0.7*	*(0.5,0.8)*	0.4	±	0.2	*0.3*	*(0.2,0.5)*	-45
Energy adjusted			0.7	±	0.3	*0.7*	*(0.6,0.9)*	0.4	±	0.2	*0.4*	*(0.3,0.5)*	-45

Eicosatrienoic (20:3n-6)	Crude	mg/day	2.8	±	0.8	*2.7*	*(2.4,3.3)*	1.4	±	0.5	*1.3*	*(1.0,1.7)*	-51
Energy adjusted			2.8	±	0.6	*2.9*	*(2.5,3.3)*	1.4	±	0.4	*1.4*	*(1.1,1.6)*	-51

Arachidonic (20:4n-6)	Crude	mg/day	10.1	±	2.3	*9.9*	*(8.8,11.1)*	5.2	±	1.5	*4.9*	*(4.2,5.9)*	-49
Energy adjusted			10.1	±	1.7	*10.0*	*(9.3,11.1)*	5.2	±	0.9	*5.2*	*(4.6,5.9)*	-49

n-6/n-3 ratio	Crude		3.7	±	0.7	*3.5*	*(3.2,4.2)*	4.0	±	1.0	*3.8*	*(3.2,4.7)*	7
Energy adjusted			3.7	±	0.7	*3.8*	*(3.2,4.2)*	4.0	±	1.0	*4.1*	*(3.2,4.7)*	7

* : 25 and 75 percentiles.

† : (FFQ mean-WDR mean)/WDR mean (%)

‡ : Total fatty acids.

§ : Saturated fatty acids.

|| : Mono unsaturated fatty acids.

¶ : Poly unsaturated fatty acids.

**: Eicosapentaenoic acid.

† † : Docosapentaenoic acids.

‡ ‡ : Docosahexaenoic acids.

Table 8 shows the Spearman correlation coefficients between WDR and FFQ. We calculated 2 kinds of coefficients, using crude nutrient intakes and energy adjusted nutrient intakes by the residual method.^8^ The Spearman correlation coefficient changed from 0.17 (linoleic acid) to 0.46 (animal fat, potassium), and the mean and median was 0.30 and 0.28, respectively. When energy adjusted nutrient intakes were used, the Spearman coefficient ranged from 0.15 (PUFA) to 0.51 (animal fat). The mean and median was 0.33 and 0.35, respectively.

**Table 8.** Spearman correlation coefficients for energy and nutrient intakes between weighed dietary record for 12-days and food frequency questionnaire.

(n=85)	Spearman correlation coefficients			

Nutrient	Crude	p value	Energy-adjusted	p value
Energy	0.20	0.065	-	-
Total protein	0.24	0.027	0.24	0.025
Animal protein	0.36	0.001	0.31	0.004
Total fat	0.28	0.010	0.46	0.000
Animal fat	0.46	0.000	0.51	0.000
Vegetable fat	0.21	0.053	0.34	0.002
Fish fat	0.28	0.008	0.21	0.049
Iron	0.26	0.018	0.28	0.009
Calcium	0.44	0.000	0.35	0.001
Potassium	0.46	0.000	0.38	0.000
Salt	0.35	0.001	0.31	0.004
Vitamin A	0.28	0.009	0.35	0.001
Vitamin B_1_	0.33	0.002	0.36	0.001
Vitamin B_2_	0.40	0.000	0.31	0.004
Vitamin C	0.38	0.000	0.27	0.012
Cholesterol	0.23	0.031	0.29	0.007
TFA*	0.26	0.018	0.39	0.000
SFA^†^	0.36	0.001	0.50	0.000
Myristic (14:0)	0.37	0.000	0.39	0.000
Palmitic (16:0)	0.33	0.002	0.51	0.000
Stearic (18:0)	0.33	0.002	0.47	0.000
MUFA^†^	0.27	0.011	0.36	0.001
Palmitoleic (16:1)	0.35	0.001	0.36	0.382
Oleic (18:1)	0.26	0.016	0.41	0.369
PUFA^§^	0.20	0.070	0.15	0.183
n-3 PUFA	0.25	0.019	0.21	0.058
Linolenic (18:3n-3)	0.22	0.044	0.16	0.152
EPA^||^ (20:5n-3)	0.26	0.015	0.19	0.089
DPA^¶^ (22:5n-3)	0.27	0.012	0.23	0.033
DHA^**^ (22:6n-3)	0.24	0.024	0.19	0.075
n-6 PUFA	0.17	0.119	0.16	0.143
Linoleic (18:2n-6)	0.17	0.120	0.16	0.154
Eicosatrienoic (20:3n-6)	0.40	0.000	0.44	0.000
Arachidonic (20:4n-6)	0.28	0.011	0.24	0.027
n-6/n-3 ratio	0.24	0.025	0.24	0.029

Mean	0.30		0.31	
Median	0.28		0.31	

* : Total fatty acids.

† : Saturated fatty acids.

‡ : Mono unsaturated fatty acids.

§ : Poly unsaturated fatty acids.

|| : Eicosapentaenoic acid.

¶ : Docosapentaenoic acids.

**: Docosahexaenoic acids.

In an epidemiologic study, researchers usually use evaluation by categorical classification rather than by the absolute value. We examined the validity of evaluating the amount of nutrient intakes by FFQ in four categories by quartiles. The percentage of coincidence (same category) varied between 45% (potassium) and 22% (MUFA). The mean coincidence percentage was 31% and the median was 33 %, and both the mean and median of the reversal category were 7%, and varied between 2% (potassium) and 11% (energy).

## DISCUSSION

In order to study the relation between chronic diseases and dietary factors, a researcher should investigate a subject’s habitual dietary intake over a long period of time, rather than during a short term. The food frequency questionnaire method (FFQ) has been developed to show habitual dietary intake.^2^ If this method is used, it will, first of all, require a validity test to verify whether it is actually capable of showing an individual’s habitual intake condition. A gold standard will be required for its validity test. We adopted the weighed dietary recording method (WDR) as the gold standard. The subjects recorded their dietary intake for 3 days every three months, totally 12 days in one year. In doing so, we took into consideration both their day-to-day variation and seasonal variation in dietary intake. Strictly speaking, subjects participating in the validity research should be chosen at random from cohort members belonging to the JACC Study. However, since WDR would be a considerable burden in terms of time and otherwise, we had to depend on volunteer members who understood the main objective of the research. Hence, the research results may not necessarily reflect the whole picture of the JACC members.

Because JACC Study was a large-scale cohort study involving more than 110,000 people, a self-administered food frequency questionnaire was used. Because the main purpose of the JACC Study was to clarify the risk factors of cancer, the questionnaire covered various areas, part of which contained questions about dietary intake. The questions were simple, mostly concerning intake frequency of a small number of food items, not their portion sizes. Second, because the focus of the questionnaire was on the kinds of food which had been considered to be the risk factors of cancer in the previous studies,^9^ we failed to evaluate the comprehensive situation regarding nutrient intake. Third, although there are ordinarily 100-150 food items are needed to evaluate the amount of nutrient intake comprehensively^2^, the research this time had only 40 items.

Considering the limited nature of the questionnaire above, we first examined the reproducibility and validity of intake frequency in detail, because frequency has a great influence on assessing the amount of nutrient intakes gained from FFQ.^2^ The mean of the percentage classified into the same category was 36%, in which the intake frequencies assessed from WDR and FFQ. When the adjoining category was included, the mean value of the percentage of coincidence was 83%. In classifying the subjects according to intake frequency, it was shown clearly, making allowance for the misclassification by the adjoining category, that most of the food items were suitable for making the category evaluation of the subjects correctly.

Although the percentage in which Japanese green tea can be classified into the same category was the highest, that of reversal misclassification was the highest as 6% (5 subjects as shown in Figure 2). Two subjects out of the five subjects were classified as “never drinking” in WDR, whereas it is classified as “drink almost every day” in FFQ. The reason this discrepancy occurred was obviously that some of those who were drinking barley tea (mugicha) in WDR, could not make a distinction between barley tea and Japanese green tea in the FFQ, and so they chose the latter beverage, thus making the percentage of green tea higher than it really was.

The percentage of coincidence of liver (any kinds) in the same category was the lowest. We thought that this was probably because most of the subjects were estimated as “never” in the category assessed by 12-day WDR, in view of the fact that people rarely eat liver daily. Even if a classification also included the adjoining category, the percentage of coincidence of the frequency between WDR and FFQ was the lowest.

As mentioned above, except for special foods, we considered the validity of intake frequency good. As for reproducibility, the correlation coefficient with the range of 0.42-0.80 was shown by this study.

We already explained that FFQ examined in this study did not show the comprehensive amount of nutrient intake because the number of question items was 40. The daily amount of energy and nutrient intake from FFQ were lower in all than those from WDR. When the difference of the amount of intake between FFQ and WDR was showed in terms of its proportion to the amount of intake from WDR, the smallest value was 15 (vitamin A) whereas the largest value was 64 (salt). We considered that the reason the difference in vitamin A was smaller than that of the other nutrients was probably because the FFQ contained three items of green and yellow vegetables, which reflect their important role as a source of vitamin A. The FFQ did not contain any seasonings as food items. According to the national nutrition survey in Japan^10^, Japanese people obtain about 70% of the daily salt intake from soy sauce, miso and such seasonings. All this explains why the difference in the amount of salt intake between FFQ and WDR was very large. Also, it is usually very difficult to evaluate the amount of seasoning intake from a FFQ.^2^^,^^11^

Tsugane and others made a detailed validity research of 138-item semi quantitative food frequency questionnaire used in Japan Public Health Center-based Prospective Study on Cancer and Cardiovascular Diseases (JPCH Study), and published a series of papers on their research results.^12^ The JPCH Study was a cohort study targeted at residents of the areas under the jurisdiction of 4 public health centers in Akita Prefecture, Iwate Prefecture, Nagano Prefecture, and Okinawa Prefecture. They chose subjects for the validity study from cohort members as volunteers. For the gold standard, they used 7-day semi-weighed dietary records four times a year (winter, spring, summer and autumn, and a total of 28 days) or 7-day dietary records two times a year (winter and spring and a total of 14 days). Sasaki obtained 102 men and 113 women as subjects and made a report about them by the sex.

Our research method was different from the JPCH validation study in that our dietary records as the gold standard were shorter and that our FFQ had only 40 items. However, our research design was similar to theirs in using the Standard Tables of Food Composition in Japan, 4th revised edition^4^ in computing nutrients. Because most of the targeted subjects were females in our study, we compared them with those in the JPCH Study. The JPCH classification was made into 5 categories by quintile, while ours was in 4 categories by quartile. The JPCH Study, which used 138 items in their FFQ, was capable of evaluating dietary intakes comprehensively. On the other hand, the correlation coefficients and analysis based on joint classification in the JACC Study were slightly lower than those of the JPCH Study. In determining portion sizes of food items, we adopted the median values of WDR, rather than general standard numerical values. This might have somehow raised the correlation coefficients between FFQ and WDR.

In conclusion, we found our FFQ suitable to deal with a large group of subjects. However, since the energy and the amount of nutrient intake from this FFQ can not show the comprehensive situation of dietary intake, subjects’ dietary intake should be assessed by categories.

## MEMBER LIST OF THE JACC STUDY GROUP

The present investigators involved, with the co-authorship of this paper, in the JACC Study and their affiliations are as follows: Dr. Akiko Tamakoshi (present chairman of the study group), Nagoya University Graduate School of Medicine; Dr. Mitsuru Mori, Sapporo Medical University School of Medicine; Dr. Yutaka Motohashi, Akita University School of Medicine; Dr. Ichiro Tsuji, Tohoku University Graduate School of Medicine; Dr. Yosikazu Nakamura, Jichi Medical School; Dr. Hiroyasu Iso, Institute of Community Medicine, University of Tsukuba; Dr. Haruo Mikami, Chiba Cancer Center; Dr. Yutaka Inaba, Juntendo University School of Medicine; Dr. Yoshiharu Hoshiyama, Showa University School of Medicine; Dr. Hiroshi Suzuki, Niigata University School of Medicine; Dr. Hiroyuki Shimizu, Gifu University School of Medicine; Dr. Hideaki Toyoshima, Nagoya University Graduate School of Medicine; Dr. Shinkan Tokudome, Nagoya City University Graduate School of Medical Science; Dr. Yoshinori Ito, Fujita Health University School of Health Sciences; Dr. Shuji Hashimoto, Fujita Health University School of Medicine; Dr. Shogo Kikuchi, Aichi Medical University School of Medicine; Dr. Akio Koizumi, Graduate School of Medicine and Faculty of Medicine, Kyoto University; Dr. Takashi Kawamura, Kyoto University Center for Student Health; Dr. Yoshiyuki Watanabe, Kyoto Prefectural University of Medicine Graduate School of Medical Science; Dr. Tsuneharu Miki, Kyoto Prefectural University of Medicine Graduate School of Medical Science; Dr. Chigusa Date, Faculty of Human Environmental Sciences, Mukogawa Women’s University ; Dr. Kiyomi Sakata, Wakayama Medical University; Dr. Takayuki Nose, Tottori University Faculty of Medicine; Dr. Norihiko Hayakawa, Research Institute for Radiation Biology and Medicine, Hiroshima University; Dr. Takesumi Yoshimura, Institute of Industrial Ecological Sciences, University of Occupational and Environmental Health, Japan; Dr. Akira Shibata, Kurume University School of Medicine; Dr. Naoyuki Okamoto, Kanagawa Cancer Center; Dr. Hideo Shio, Moriyama Municipal Hospital; Dr. Yoshiyuki Ohno, Asahi Rosai Hospital; Dr. Tomoyuki Kitagawa, Cancer Institute of the Japanese Foundation for Cancer Research; Dr. Toshio Kuroki, Gifu University; and Dr. Kazuo Tajima, Aichi Cancer Center Research Institute.

## APPENDIX 1.

For each food listed, circle the number in the box indicating how often, you have used.

**Table:**

	Food	Average use				

		Almost never	1-2 per month	1-2 per week	3-4 per week	Almost every day
1	Beef	1	2	3	4	5
2	Pork (excluding ham or sausage)	1	2	3	4	5
3	Ham or sausage	1	2	3	4	5
4	Chicken	1	2	3	4	5
5	Liver	1	2	3	4	5
6	Eggs	1	2	3	4	5
7	Milk	1	2	3	4	5
8	Yogurt	1	2	3	4	5
9	Cheese	1	2	3	4	5
10	Butter	1	2	3	4	5
11	Margarine	1	2	3	4	5
12	Deep-fried foods or tempra	1	2	3	4	5
13	Fried vegetables	1	2	3	4	5
14	Fresh fish (sashimi; sliced raw fish, boiled fish or broiled fish)	1	2	3	4	5
15	Kamaboko(fish paste)	1	2	3	4	5
16	Dried fish or salted fish	1	2	3	4	5
Green and yellow vegetables						
17	Green leaves vegetables (e. g. spinach, garland chrysanthemum, etc.)	1	2	3	4	5
18	Carrot or pumpkin	1	2	3	4	5
19	Tomatoes	1	2	3	4	5
Other types of vegetables						
20	Cabbage or head lettuce	1	2	3	4	5
21	Chinese cabbage	1	2	3	4	5
22	Edible wild plants(e.g. blacken or flowering fern)	1	2	3	4	5
23	Fungi(enokidake, shiitake mushroom)	1	2	3	4	5
24	Potatoes(e.g. white potato or sweet potato)	1	2	3	4	5
25	Algae(seaweeds)	1	2	3	4	5
26	Pickles (e. g. takuwan, hakusaiduke, etc.)	1	2	3	4	5
27	Preserved foods using soy sauce	1	2	3	4	5
28	Boiled beans	1	2	3	4	5
29	Tofu(soybean curd)	1	2	3	4	5
30	Citrus fruits	1	2	3	4	5
31	Fresh fruits juice (in summer season)	1	2	3	4	5
32	Other fruits (excluding citrus fruits).	1	2	3	4	5
33	Sweets (e.g. manjyu, yokan,cake,etc.)	1	2	3	4	5
